# Supplementary material for: Patient Education and Communication in Palliative Radiotherapy: A Narrative Review
Source: Cancers (Basel). 2025 Sep 24;17(19):3109. doi: 10.3390/cancers17193109 (PMC12524152; doi:10.3390/cancers17193109)
Supplement: Supplementary file 1 [file cancers-17-03109-s001.zip › cancers-3873115-supplementary.pdf]

Supplementary Method S1 - Database-Specific Search Strategies (search close: 18 July 2025)

SEARCH STRATEGY

PubMed

(("Radiotherapy"[Mesh] OR radiotherap*[tiab] OR "radiation therapy"[tiab] OR "external beam"[tiab]) AND ("Palliative Care"[Mesh] OR palliat*[tiab] OR "best supportive care"[tiab] OR "end of life"[tiab])) AND ("Patient Education as Topic"[Mesh] OR "Health Education"[Mesh] OR educat*[tiab] OR counsel*[tiab] OR "self-management"[tiab] OR psychoeducation[tiab] OR "decision aid*"[tiab] OR coach*[tiab]) AND (randomized controlled trial[pt] OR controlled clinical trial[pt] OR randomi?ed[tiab] OR randomly[tiab] OR trial[ti]) NOT (animals[mh] NOT humans[mh])

SCOPUS

(TITLE-ABS-KEY ((radiotherap* OR "radiation therapy" OR "external beam") AND (palliat* OR "palliative care" OR "best supportive care" OR "end of life"))) AND (TITLE-ABS-KEY (educat* OR counsel* OR "self-management" OR psychoeducation OR "decision aid*" OR coach*)) AND (INDEXTERMS ("randomized controlled trial") OR TITLE-ABS-KEY (randomized W/3 control* W/3 trial) OR TITLE-ABS-KEY (randomly W/2 assign*)) AND NOT (TITLE-ABS-KEY (animal*) OR TITLE-ABS-KEY (murine) OR TITLE-ABS-KEY (mouse) OR TITLE-ABS-KEY (rat)) AND DOCTYPE (ar) AND SRCTYPE (j)

Cochrane library

((radiotherap* OR "radiation therapy" OR "external beam") AND (palliat* OR "palliative care" OR "best supportive care" OR "end of life")):ti,ab,kw AND (educat* OR counsel* OR "self-management" OR psychoeducation OR "decision aid*" OR coach*):ti,ab,kw AND ("randomized controlled trial":pt OR (randomi?ed NEAR/3 control* NEAR/3 trial):ti,ab,kw OR (randomly NEAR/2 assign*):ti,ab,kw) NOT (animal* OR murine OR mouse OR rat):ti,ab,kw

Supplementary Figure S1

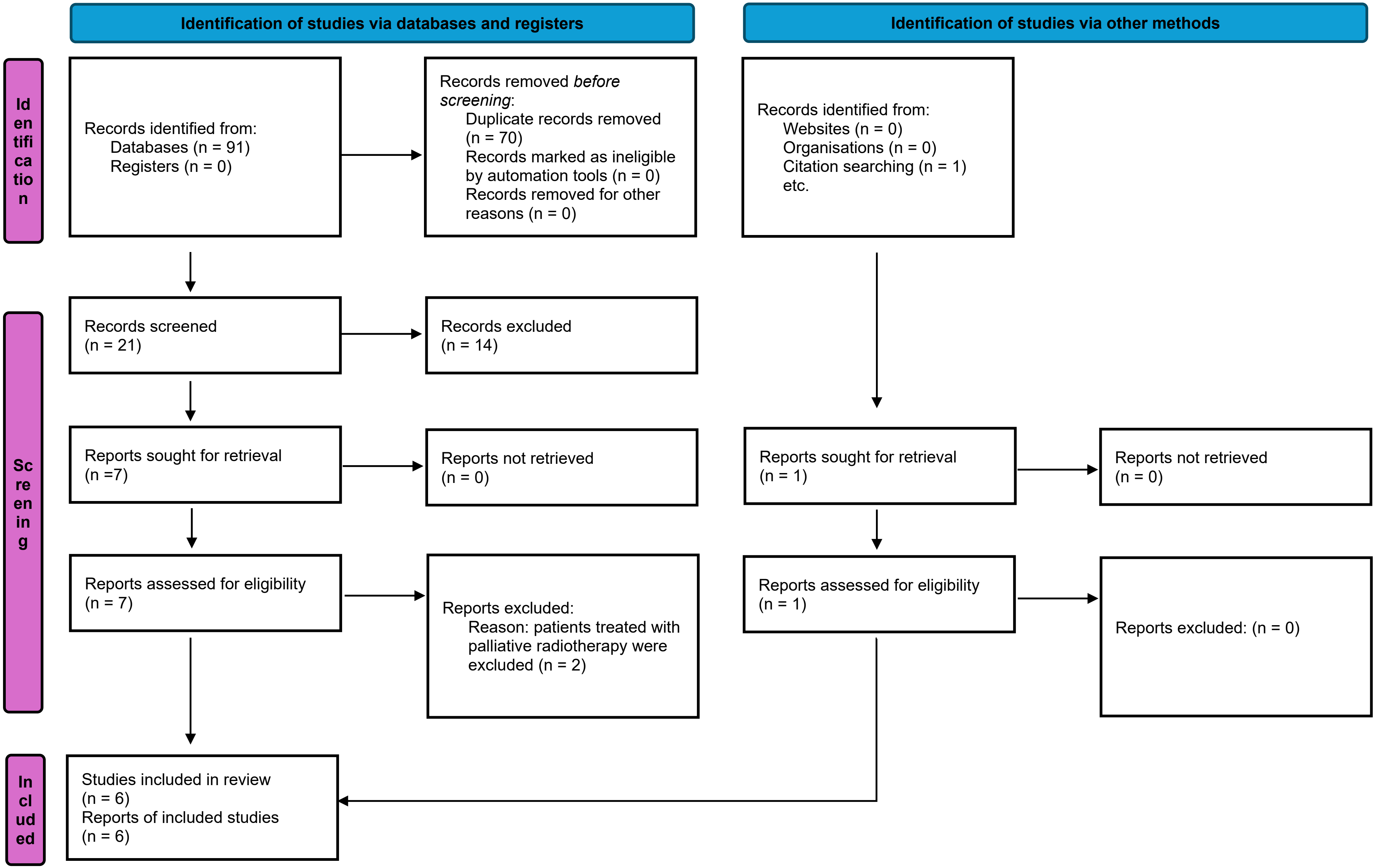

Supplementary Table S1: Full-text articles excluded after eligibility assessment

#	Full citation	Reason for exclusion
1	Charalambous A, Molassiotis A, Summers Y, Stamataki Z, Taylor P. Use of inspiratory muscle training in managing dyspnoea in lung cancer patients. Journal of thoracic oncology 2017; 12:S206-S208.	Patients receiving palliative radiotherapy were explicitly excluded by protocol
2	Vallières I, Aubin M, Blondeau L, Simard S, Giguère A. Effectiveness of a clinical intervention in improving pain control in outpatients with cancer treated by radiation therapy. Int J Radiat Oncol Biol Phys. 2006 Sep 1;66(1):234-7.	Patients receiving palliative radiotherapy were explicitly excluded by protocol
